# Potential Pitfalls on the ^99m^Tc-Mebrofenin Hepatobiliary Scintigraphy in a Patient with Biliary Atresia Splenic Malformation Syndrome

**DOI:** 10.3390/diagnostics6010005

**Published:** 2016-01-07

**Authors:** Jane Maestri Brittain, Lise Borgwardt

**Affiliations:** 1 Department of Clinical Physiology, Nuclear Medicine & PET, Rigshospitalet–Glostrup, Copenhagen University Hospital, Nordre Ringvej 57, DK-2600 Glostrup, Denmark; 2 Department of Clinical Physiology, Nuclear Medicine & PET, Rigshospitalet, Copenhagen University Hospital, Blegdamsvej 9-KFNA 4011, DK-2100 Copenhagen, Denmark; lise.borgwardt@regionh.dk

**Keywords:** biliary atresia, biliary atresia splenic malformation syndrome, hepatobiliary scintigraphy, magnetic resonance imaging

## Abstract

Biliary atresia (BA) is an obliterative cholangiopathy affecting 1:10.000–14.000 of newborns. Infants with Biliary Atresia Splenic Malformation syndrome (BASM) are a subgroup of BA patients with additional congenital anomalies. Untreated the disease will result in fatal liver failure within the first years of life. Kasai portoenterostomy restores bile flow and delay the progressive liver damage thereby postponing liver transplantation. An early diagnosis is of most importance to ensure the effectiveness of the operation. The ^99m^Tc-Mebrofenin hepatobiliary scintigraphy is part of the diagnostic strategy when an infant presents jaundice due to conjugated hyperbilirubinemia (>20 µmol/L total bilirubin of which 20% is conjugated) with its high sensitivity of 97%–100% in refuting BA. Rapid extraction of tracer by the liver and no visible tracer in the small bowl after 24 h is indicative of BA. Laparotomy with antegrade cholangiography is then performed giving the final diagnosis when the remains of the obliterated biliary tree are revealed in the case of BA. We present a case demonstrating some of the challenges of interpreting the ^99m^Tc-Mebrofenin hepatobiliary scintigraphy in an infant with BASM and stress the importance that the ^99m^Tc-Mebrofenin hepatobiliary scintigraphy is part of a spectrum of imaging modalities in diagnosing BA.

**Figure 1:**
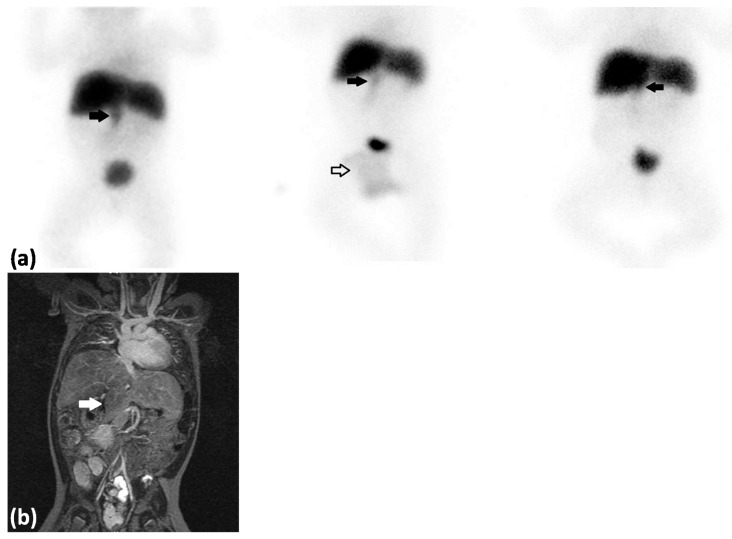
(**a**) ^99m^Tc-Mebrofenin hepatobiliary scintigraphy anterior view—**left**: summarized dynamic images 52–56 min after tracer injection; **middle**: static image 2 h after tracer injection; **right**: static image 5 h after tracer injection. A 21 day old girl with known situs inversus of the stomach diagnosed in relation to an operation for malrotation and duodenal atresia who presented with jaundice. Laboratory work-up showed conjugated hyperbilirubinemia (total bilirubin, 239 µmol/L (normal range 4–17 µmol/L), conjugated bilirubin, 191 µmol/L (normal range <4 µmol/L)), elevated alanine aminotransferase (ALT, 57 U/L (normal range 10–45 U/L)), elevated aspartate aminotransferase (AST, 94 U/L (normal range 15–65 U/L)), normal alkaline phosphatase (ALP, 415 U/L (normal range 55–425 U/L)), and elevated gamma-glutamyl transpeptidase (GGTP, 1021 U/L (normal range 10–130 U/L)). Biliary atresia (BA) was suspected and the patient was referred for a ^99m^Technetium-trimethylbromo-iminodiacetic acid hepatobiliary scintigraphy (^99m^Tc-Mebrofenin hepatobiliary scintigraphy) which showed rapid extraction of tracer by the liver with no excretion to the small bowl but with suspicion of visualization of the gallbladder (thick black arrows). Tracer was physiologically excreted with the urine and localized in a diaper (white arrow). Rapid tracer extraction by the liver is indicative of normal functioning hepatocytes which can be expected in BA and in part rule out neonatal hepatitis which is a differential diagnosis to BA in infants with conjugated hyperbilirubinemia. No excretion of tracer to the small bowl indicates BA but visualization of the gallbladder is not common in BA since the common bile duct is affected in the majority of cases [1]. Because of the known altered anatomy and the somewhat unexpected result of the ^99m^Tc-Mebrofenin hepatobiliary scintigraphy it was decided to perform a laparotomy at this stage. It was not possible to perform an antegrade cholangiography since the biliary tree including the gallbladder was atrophic. The patient was diagnosed with Biliary Atresia Splenic Malformation syndrome (BASM) and underwent a Kasai portoenterostomy. The hepatobiliary scintigraphy study indeed helped to diagnose BA but it could not be the atrophic gallbladder that was visualized caudally from the liver. Because of disease progression into biliary cirrhosis a liver transplantation nine month later was indicated. Prior to the liver transplantation a magnetic resonance imaging (MRI) of the abdomen was performed; (**b**) MRI, coronal view (T2 weighted), which demonstrated that the liver was separated into several lobes. One of these hepatic lobes (white arrow) was localized caudally and was the structure interpreted as the gallbladder on the ^99m^Tc-Mebrofenin hepatobilliary scintigraphy. Furthermore the MRI showed that the inferior vena cava was localized in the left side of the abdomen and confirmed the known situs inversus of the stomach. Patients with BASM can present different malformations including asplenia, double spleen, polysplenia, or a normal spleen, situs inversus and malrotation of viscera, malformation of the intraabdominal veins and structural cardiac anomalies [2,3]. This case illustrates the challenges of interpretation of the ^99m^Tc-Mebrofenin hepatobiliary scintigraphy when the anatomy is altered. Care must be taken to avoid misinterpreting the scintigraphic findings; in particular, anomalous liver anatomy can be mistaken for the gallbladder if one is not careful. The ^99m^Tc-Mebrofenin hepatobiliary scintigraphy should always be part of a spectrum of imaging modalities in the diagnostic strategy of BA.
